# Why More Psychological Therapy Would Cost Nothing

**DOI:** 10.3389/fpsyg.2015.01713

**Published:** 2015-11-25

**Authors:** Richard Layard, David M. Clark

**Affiliations:** ^1^Centre for Economic Performance, The London School of Economics and Political ScienceLondon, UK; ^2^Department of Experimental Psychology, Medical Sciences Division, University of OxfordOxford, UK

**Keywords:** psychological therapy, cost-effectiveness, evidence-based psychological therapies, mental health burden, psychotherapy

To tackle the huge problem of mental illness, England has launched a large programme of psychological therapy, which is being watched worldwide. The authors argue that it costs nothing, due to savings on welfare benefits and physical healthcare. The article is based on the recent book *Thrive: The power of evidence-based psychological therapies* (Layard and Clark, 2014b).

In rich countries 38% of all illness is mental illness (p. 43–45, Layard and Clark, 2014b). It particularly affects people of working age where it accounts for 50% of the total (see Figure 1). The overall economic cost has been estimated at 8% of GDP, not to mention the massive suffering involved. Policy-makers increasingly wonder what they can do about it.

**Figure 1:**
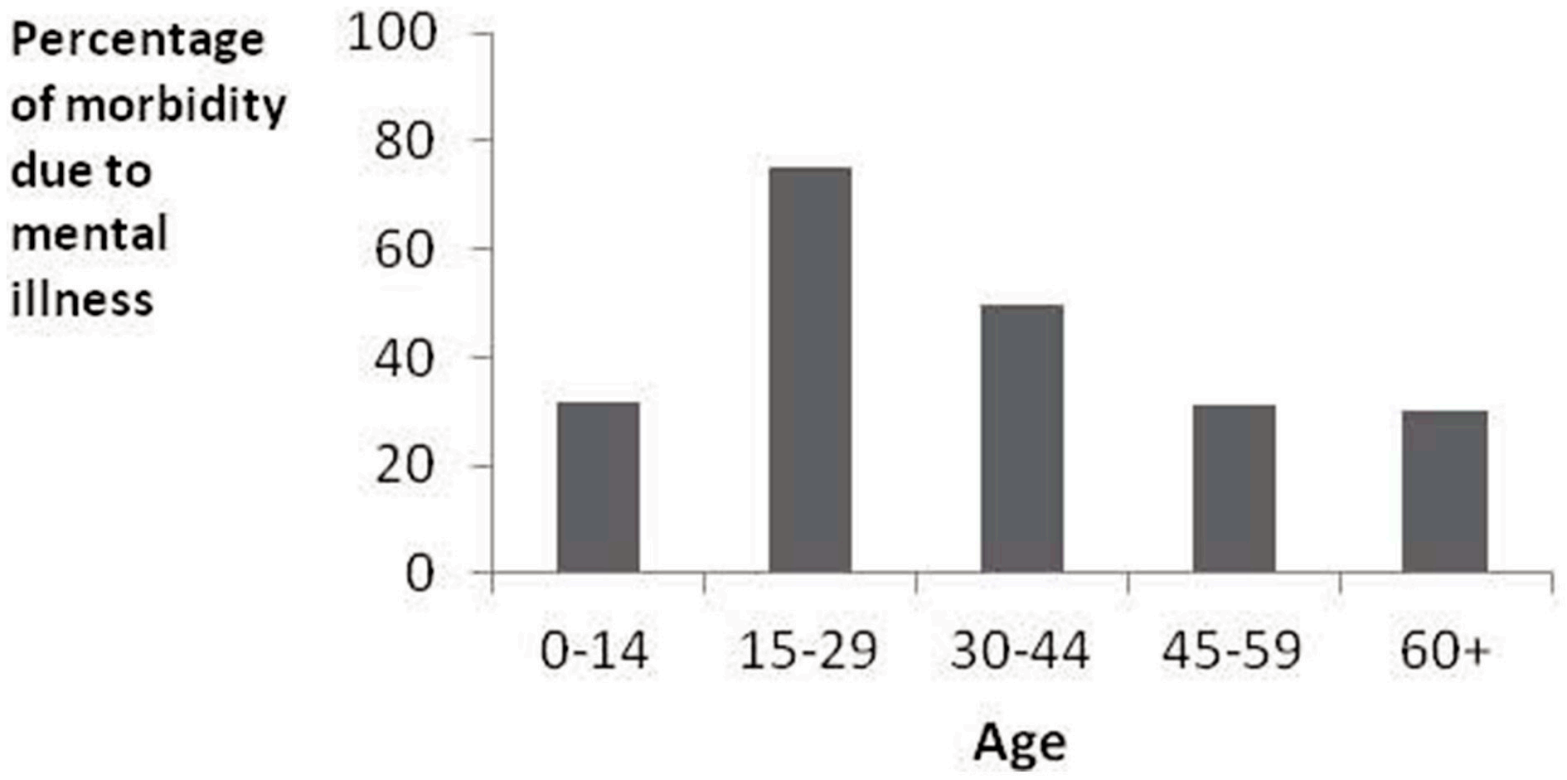
**Mental illness is the main health problem of working age in rich countries**.

Fortunately the last 40 years have seen huge progress in evidence-based psychological therapies especially cognitive-behavioral therapy (CBT). For people with clinical depression or chronic anxiety disorders this leads to 50% recovery rates, with many others also improving substantially. It also halves the likelihood of relapse; in this respect it is more effective than drugs. It is also what the great majority of patients would prefer (Mchugh et al., 2013).

Yet in most countries only a tiny minority of people with depression or anxiety disorders get evidence-based psychological therapy, meaning a therapy supported by the Cochrane Collaboration or Britain's National Institute for Health and Care Excellence (NICE). In Britain in 2007 one in six people (surveyed in their homes) met the diagnostic criteria for depression or anxiety disorders like OCD, PTSD, panic disorder or social anxiety disorder. Of those, 1% received evidence-based psychological therapy. The situation is much better today in England thanks to a major programme of training and service development called Improving Access to Psychological Therapies (IAPT) which began in 2008. By now it is assessing about 13% of the diagnosable population, and treating about 8%—nearly half a million people. It has been called world-beating in the journal *Nature*. But still the whole country is not covered and waiting times are dangerously long. The authors and many others are arguing that the programme needs to double by 2020.

A central issue has always been cost, and one appeal of the programme to policy-makers has been the cost savings which it generates. Averaged over all patients from mild to severe and all lengths of treatment (from those who drop out after two sessions to those who get up to 20 sessions), the cost per patient is £650. Against this we have to set the savings on, first, welfare benefits, and then physical healthcare.

As we have mentioned, mental illness is the main illness of working age. In most rich countries about 1% of the working age population are on disability benefits due to depression or anxiety disorders. In Britain one such person costs the government £650 a month more than if they were not on benefit (This includes both the benefits and reduced tax payments; Department for Work Pensions, 2014^1^). So suppose we treat a representative group of people with depression or anxiety disorders. If as a result of treatment 4% of those treated worked an extra 25 months, the average patient would be working 1 month more than otherwise. This would be enough to repay the cost of the treatment.

Is 4% a realistic estimate? A number of randomized trials in the US and Britain come up with much larger numbers (Layard and Clark, 2014b). So, more psychological therapy is hugely in the interest of Finance Ministries worldwide.

But on top of this there is another equally important area of savings—on physical healthcare. Suppose we take 2 people with the same type and degree of physical illness, but A also has depression or an anxiety disorder. Then A will get around 50% more physical healthcare than B—partly because of the physiological effects of mental illness, partly because of unhealthy habits, and partly due to extra levels of anxiety. Figure 2 shows some striking figures on the effects of depression from the Colorado Access insurance scheme.

**Figure 2:**
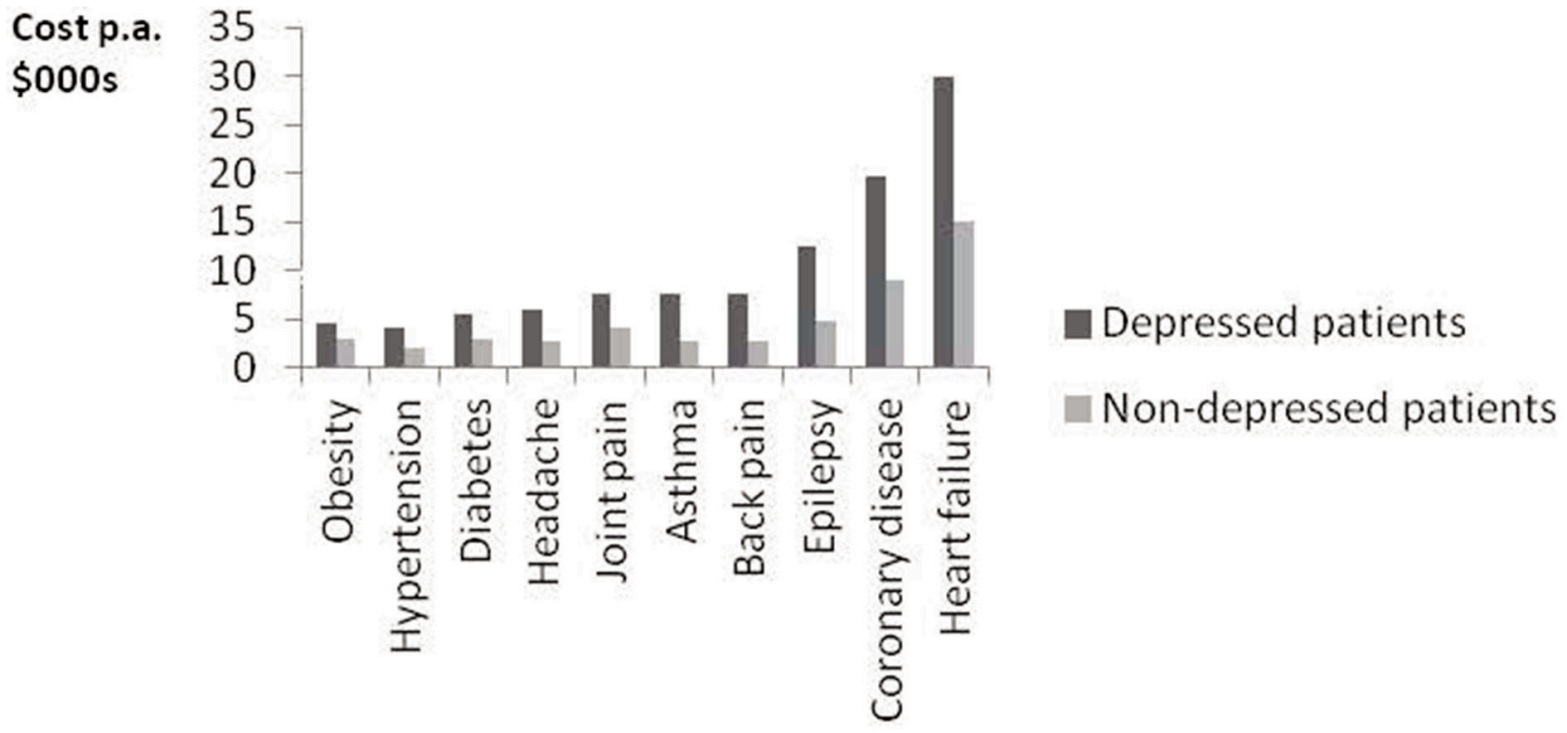
**Depression increases the cost of physical healthcare**.

In Britain the cost of physical healthcare is around £2000 extra when the patient is also mentally ill. So if we treat a physically ill person for their mental illness we can expect to save up to £1000 a year on physical healthcare (due to the 50% recovery rate). One enterprising British general medical practice has in fact tracked the physical healthcare costs of its mentally ill patients and found similar results. They tracked all their mentally ill patients and compared those who had been treated by the IAPT programme with those who had not. The difference in physical healthcare costs was about £750 a year. This compares with the one-off cost of £650 for the psychological therapy.

Of course not all mentally ill people have physical problems, though over 50% do. But in England the biggest future expansion of psychological therapy will be among people who also have physical problems. In such cases the savings are even greater if the psychological therapy is explicitly tailored to take into account the physical problem—such as breathlessness, heart pain or back pain. Numerous innovations of this kind have shown huge savings in the cost of physical healthcare—often up to four times the cost of the psychological therapy (Gulliksson et al., 2011). For example, Figure 3 shows the progress of Swedish patients discharged from hospital following a heart attack. One set of patients was given group CBT in 20 sessions over a year, the other was given none.

**Figure 3:**
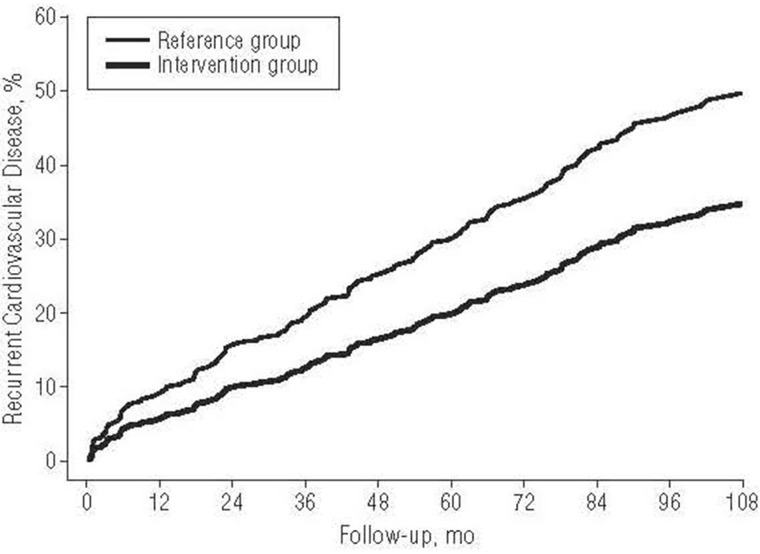
**CBT reduces the recurrence of cardiovascular disease**. All readings are adjusted for initial medical condition.

We can summarize our cost findings in Table 1. In most countries the savings in benefits and taxes accrue to the public finances, and may be of little interest to those who finance healthcare. But the healthcare savings accrue to exactly the same authorities as finance the psychological therapy (in Britain the National Health Service and in most other countries the health insurance system).

**Table 1:** **Costs and savings from psychological therapy (England)**.

**Cost**	**£650 (one-off)**
Savings in welfare benefits and extra taxes	> £650 (within 2 years)
Savings in reduced physical healthcare	> £650 (per year, duration unknown)

Against this background the case for expanding psychological therapy is surely overwhelming. It responds to a huge problem. It is effective. It would cost nothing to the system. And it would relieve a mass of suffering (Layard and Clark, 2014a).

## Author note

This contribution is a modified version of the article “Why more psychological therapy would cost nothing” [Online] reproduced with permission from http://www.voxeu.org/article/psychological-therapy-costs-nothing: VOX CEPR's Policy Portal [Accessed 17 July 2014].

### Conflict of interest statement

The authors declare that the research was conducted in the absence of any commercial or financial relationships that could be construed as a potential conflict of interest.

## Footnotes

^1^Available online at: https://www.gov.uk/government/organisations/department-for-work-pensions
